# Deep sequencing of HPV E6/E7 genes reveals loss of genotypic diversity and gain of clonal dominance in high-grade intraepithelial lesions of the cervix

**DOI:** 10.1186/s12864-017-3612-y

**Published:** 2017-03-14

**Authors:** Jane Shen-Gunther, Yufeng Wang, Zhao Lai, Graham M. Poage, Luis Perez, Tim H. M. Huang

**Affiliations:** 1 Department of Clinical Investigation, Brooke Army Medical Center, Gynecologic Oncology & Clinical Investigation, 3698 Chambers Pass, Fort Sam Houston, TX 78234 USA; 2 Department of Biology, University of Texas at San Antonio, San Antonio, TX 78249 USA; 3 Greehey Children’s Cancer Research Institute, University of Texas Health Science Center at San Antonio, San Antonio, TX 78229 USA; 4 Department of Molecular Medicine, Cancer Therapy and Research Center, University of Texas Health Science Center at San Antonio, San Antonio, TX 78229 USA

**Keywords:** Human papillomavirus, HPV genotyping, High-throughput sequencing, Deep sequencing, Metagenome, Virome, LSIL, HSIL

## Abstract

**Background:**

Human papillomavirus (HPV) is the carcinogen of almost all invasive cervical cancer and a major cause of oral and other anogenital malignancies. HPV genotyping by dideoxy (Sanger) sequencing is currently the reference method of choice for clinical diagnostics. However, for samples with multiple HPV infections, genotype identification is singular and occasionally imprecise or indeterminable due to overlapping chromatograms. Our aim was to explore and compare HPV metagenomes in abnormal cervical cytology by deep sequencing for correlation with disease states.

**Results:**

Low- and high-grade intraepithelial lesion (LSIL and HSIL) cytology samples were DNA extracted for PCR-amplification of the HPV E6/E7 genes. HPV+ samples were sequenced by dideoxy and deep methods. Deep sequencing revealed ~60% of all samples (*n* = 72) were multi-HPV infected. Among LSIL samples (*n* = 43), 27 different genotypes were found. The 3 dominant (most abundant) genotypes were: HPV-39, 11/43 (26%); -16, 9/43 (21%); and -35, 4/43 (9%). Among HSIL (*n* = 29), 17 HPV genotypes were identified; the 3 dominant genotypes were: HPV-16, 21/29 (72%); -35, 4/29 (14%); and -39, 3/29 (10%). Phylogenetically, type-specific E6/E7 genetic distances correlated with carcinogenic potential. Species diversity analysis between LSIL and HSIL revealed loss of HPV diversity and domination by HPV-16 in HSIL samples.

**Conclusions:**

Deep sequencing resolves HPV genotype composition within multi-infected cervical cytology. Biodiversity analysis reveals loss of diversity and gain of dominance by carcinogenic genotypes in high-grade cytology. Metagenomic profiles may therefore serve as a biomarker of disease severity and a population surveillance tool for emerging genotypes.

**Electronic supplementary material:**

The online version of this article (doi:10.1186/s12864-017-3612-y) contains supplementary material, which is available to authorized users.

## Background

The first description of cervical cancer was documented by Hippocrates c. 450 BCE [1]. Its cause remained a mystery for two millennia until 1983 when zur Hausen and colleagues isolated and cloned HPV-16 from cervical carcinoma [2]. HPV is now recognized as the carcinogen of almost all invasive cervical cancer and a major cause of other human malignancies including vulvovaginal, oropharyngeal, penile, and anal cancers [3, 4].

The HPV genome is a ~8,000 base pair (bp), double-stranded, circular DNA. The prototypical genome encodes 6 early genes (E1, E2, E4, E5, E6, and E7) and 2 late genes (L1 and L2) [5]. By convention, HPV classification is based on the L1 gene where a difference of >10% in the viral sequence defines a different genotype [6]. With the advent of sequencing technologies, the list of papillomavirus (PV) genotypes has grown to 333 with 202 types isolated from humans and 131 from animals [7]. The conferment of HPV oncogenic potential is derived from two viral oncoproteins E6 and E7, which inactivate two primary cellular proteins: p53 and RB, respectively [5, 8, 9]. Inhibition followed by degradation of p53 and RB leads to cell-cycle progression, immortalization, and malignant transformation of the HPV-infected cell [5]. The E6 and E7 proteins of carcinogenic HPV possess a structural advantage, namely, conformational plasticity that may be responsible for multi-target binding and transformation in host cells [9, 10]. Hence, sequencing the E6/E7 genes to decode its oncogenic potential through association with known genotypes by genetic proximity may be a preferred diagnostic test.

Sanger sequencing of PCR amplicons is accurate in the detection of single HPV infections and yields easily interpretable data [11]. For samples with multiple HPV infections, genotype identification may be imprecise or indeterminable because of noisy (overlapping signals) chromatograms causing failures in nucleotide alignment [11]. Furthermore, non-dominant genotypes in mixed infections may not be detected by the Sanger method and may be consequently underestimated. Therefore, to properly identify all HPV types in a complex sample, contemporary next-generation sequencing (NGS), also referred to as deep or high-throughput sequencing (HTS), may provide an innovative solution [12]. Although the literature is limited, several NGS platforms have been successful at determining the diversity of HPV genotypes found in human skin, normal cervical cytology, and cytology of an HIV+ woman harboring 16 types [13–16].

In this study, we aimed to determine the HPV genotypes and their proportional composition in single- and multi-infected cervical samples. Specifically, two categories of cytology, i.e. low- and high-grade squamous intraepithelial lesion (LSIL and HSIL) containing HPV DNA were selected for deep (Illumina®) sequencing to explore viral diversity and characterize differences in metagenomes.

## Methods

### Subjects and samples

This study was conducted after approval by the Institutional Review Board of Brooke Army Medical Center (BAMC), Texas. Liquid-based cytology collected for clinical testing at the Department of Pathology was consecutively procured after completion of analysis for cytological diagnosis. Demographic data were abstracted from the electronic health record (AHLTA) of the Department of Defense (DoD) and linked to each specimen. Similarly, histologic data were abstracted for cytohistological correlation. In a previous study, three categories of samples, i.e. negative for intraepithelial lesion or malignancy (NILM), LSIL and HSIL were collected for HPV genotyping and DNA methylation analysis [17]. For this study, we selected only the subset of HPV+ LSIL (*n* = 55) and HSIL (*n* = 29) for characterization and comparison of viral diversity because of the high prevalence of HPV positivity.

### HPV DNA amplification and detection

**Fig. 1:**
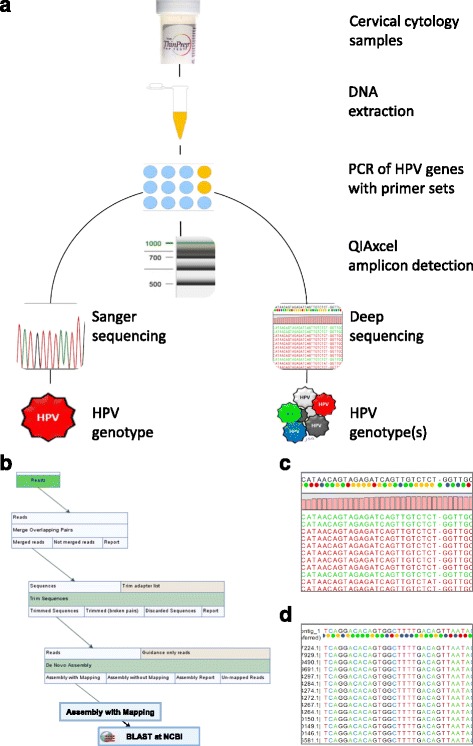
Protocol schema and bioinformatics workflow. **a** Residual liquid-based cervical cytology collected for DNA extraction, PCR amplification of HPV DNA, and amplicon detection by capillary electrophoresis. Samples with + HPV DNA were sequenced by dideoxy (Sanger) and deep sequencing (Illumina®) methods for genotype identification. **b** Bioinformatics workflow created for next-generation sequencing (NGS) data and BLAST® search for HPV genotyping. Workflow layout in CLC Genomics Workbench consisting of 5 sequential steps: reads import, reads merging, reads QC, de novo assembly with mapping, and NCBI BLAST® search. **c** Representative read mapping result. Top to bottom: consensus sequence with nucleotide color space encoding (colored dots), coverage level (pink-bar height), and sequence reads (forward: green; reverse: red). **d** Representative nucleotide BLAST® result. The top sequence is the input (query) sequence, e.g., a consensus sequence derived from de novo assembly with nucleotide encoding (colored dots). Below, the matched (hit) sequences are displayed with nucleotide coloring and GenBank ID on the left. BLAST, Basic Local Alignment Search Tool; NCBI, National Center for Biotechnology Information; QC, Quality Control

Cervical cytology (10 mL) was centrifuged (4,000 rpm x 2 min), and the supernatant was removed (laboratory schema shown in Fig. 1a). The cell pellet (200–250 uL) was transferred into sample tubes for DNA extraction using the QIAamp DNA Mini kit on the QIAcube robot (QIAGEN). The purified DNA in 150 uL of eluent was quantified by spectrophotometry and stored at -20 °C. For HPV DNA amplification, the consensus primer set: GP-E6-3 F/GP-E7-5B/GP-E7-6B was used to amplify a region of E6/E7 genes for genotype identification [18, 19]. The Multiplex PCR Plus kit (QIAGEN) was used with the triplet primer set per manufacturer’s instructions. Briefly, PCRs were performed in a final volume (50 uL) containing template DNA (200 ng), PCR master mix (25 uL), forward and reverse primers (1uM each, final concentration), and RNAase-free water. The cycling protocol was as follows: activation [95 °C x 5 min]; 45 cycles [94 °C x 30 s, 55 °C x 90 s, 72 °C x 90 s]; final extension [72 °C x 10 min]. After PCR, high-resolution capillary gel electrophoresis was used to detect amplicons by the QIAxcel (QIAGEN) with a detection sensitivity of 0.1 ng/μL and DNA resolution of 3–5 bp. Samples with ≥1 amplicon were sequenced.

### HPV DNA dideoxy (Sanger) sequencing and genotyping

PCR products were purified using the GeneRead Size Selection Kit (QIAGEN) and eluted in 100 uL of molecular biology-grade water on the QIAcube. Dideoxy sequencing of the amplicons (~200 ng DNA/sample) was performed using primer GP-E6-3 F at Eurofins Operon (USA). Sequence quality was assessed using Sequence Scanner 2.0 (appliedbiosystems.com) where a “high quality” Trace Score (TS) was defined as ≥20 and a QV20+ value (total number of bases in the sequence with TS ≥20) as ≥100. Quality sequences were entered into BLAST^®^ and queried against HPV sequences in GenBank^®^ (Taxon identifier: 151340) for genotyping as previously described [11].

### HPV DNA sample library preparation and deep sequencing

DNA libraries were prepared from GeneRead-purified PCR products as described above using the Nextera XT kit (Illumina). Briefly, the input DNA was quantitated and analyzed for purity (260/280 nm absorbance ratio ~1.8-2.0) with the Qubit Fluorometer (ThermoFischer). Each DNA sample (1 ng) with a standardized concentration of 0.1-0.2 ng/uL was “tagmented” (fragmented and tagged with sequencing adapters) by the Nextera XT transposome and dual indexed (barcoded) by limited-cycle PCR using the 96-sample Nextera Index Kit. AMPure magnetic beads (Beckman Coulter) were used to purify the DNA libraries and size select (300–500 bp) amplicons in each sample. The DNA libraries were normalized for quantity to ensure equal representation from each sample prior to pooling and sequencing. Paired-end bi-directional sequencing (2 × 300 bp) using the MiSeq Reagent Kit v3 (600-cycle) was performed on the MiSeq (Illumina) for bridge amplification.

### Bioinformatics for next-generation genotyping

The MiSeq on-instrument analysis generated a QC report of total reads, total reads passing filter, and % of bases with quality score ≥30 (Q30) meaning an accuracy rate of 99.9% [20]. The de-multiplexed, paired-end sequences were imported into CLC Genomics Workbench 8.0 (QIAGEN) for analysis. The bioinformatics workflow constructed for HPV genotyping consisted of 5 sequential steps: reads import, reads merging, reads QC, de novo assembly with mapping, and BLAST^®^ search (Fig. 1b). A read mapping result following de novo assembly produced contigs and the consensus sequence (Fig. 1c). Only consensus sequences with contigs composed of ≥100 reads from each sample were BLAST^®^ (blastn) searched against the NCBI Viral Genome database. BLAST^®^ hit results (Fig. 1d) were used for HPV genotype assignment [11]. Of note, the minimum coverage and percentage of reads required for accurate HPV genotype identification has not been reported to date. For this study, the minimum coverage (100x) for variant detection was based on the manufacturer’s technical note on coverage requirement for reads mapped to a subset of a genome [21]. The CLC Genomics workflow parameter settings are presented in Additional File 1: Table S1.

### Species diversity and phylogenetic analysis

The term *species* in biodiversity analysis refers to a sampling unit under study. Herein, the sample unit is the HPV *genotype* rather than *species.* HPV *community* is defined as the assemblage of different genotypes found in each sample by deep sequencing and grouped as LSIL or HSIL for comparative analysis. Count-based genotype diversity and dominance within a HPV community were quantified by the Shannon-Wiener Index (SWI) and Berger-Parker Index (BPI), respectively and compared using Solow’s randomization test [22]. Composition-based genotype dissimilarity between communities (β-diversity) was calculated using the Analysis of Similarity (ANOSIM) method based on percentage differences between samples [23, 24]. ANOSIM is a non-parametric statistical test for significant differences in species composition (%) between two or more groups/sites of sampling units. The ANOSIM statistic *R* compares the mean ranks of species similarities between and within groups [24]. Principal component analysis (PCA) was used to determine the most influential variables (HPV types) in the LSIL or HSIL group. PCA was performed on the covariance matrix of natural log-transformed abundance data [ln(x + 1)] of HPV-types within each sample [24, 25]. Log transformation was applied to reduce the influence of highly abundant genotypes (skewed data). Biodiversity analyses were performed using Species Diversity and Richness 4.0 and Community Analysis Package 5.0. [22, 24]

The evolutionary relationship of HPV E6/E7 from deep sequencing of LSIL and HSIL samples was inferred using the Neighbor-Joining method [26]. The evolutionary distances were computed using the Maximum Composite Likelihood method [27]. Codon positions included were 1st + 2nd + 3rd + Noncoding. Positions containing gaps or missing data were eliminated. Bootstrap analysis using 1,000 replicates was performed to evaluate the reliability of the inferred trees [28]. The bootstrap value attached to each node is the confidence (%) in the subtree rooted at the node. Evolutionary analyses were conducted in MEGA6 [29].

### Definitions

The classification of HPV carcinogenic potential was based on the World Health Organization (WHO) International Agency for Research on Cancer (IARC) Working Group Reports [8, 30]. Specifically, HPV types 16, 18, 31, 33, 35, 39, 45, 51, 52, 56, 58, 59, and 68 were deemed *carcinogenic*; HPV types 26, 30, 34, 53, 66, 67, 69, 70, 73, 82, 85, and 97 were *possibly carcinogenic*; HPV types 6, 11 were *not classifiable*; and all others were *probably not carcinogenic*. The *not classifiable* agents are generally considered not carcinogenic based on limited epidemiological and experimental data. The rationale for categorizing HPV 6 as *not classifiable* instead of *probably not carcinogenic* was the low (0.45% [95% CI: 0.35-0.56]) but not zero incidence found in invasive cervical cancers worldwide [30]. It is postulated that HPV-6 and other low-risk genotypes may rarely cause cancer due to unusual “virus-host circumstances” [30]. Therefore, the *not classifiable*/*probably not carcinogenic* genotypes are generally considered non-carcinogenic and thus grouped together for this study. The *probably not carcinogenic* group include HPV species alpha-1, -2, -3, -4, -8, -10 (other than HPV 6, 11), -13, -14/15 [8, 30].

### Statistical analysis

Data were summarized using means (95% CI), medians (IQR), and proportions. Normality of data distribution was determined by the skewness and kurtosis test. For nonparametric data, the median test (Fisher’s exact, 2-tailed) or chi-squared test were used for hypothesis testing as appropriate. Agreement between 2 dichotomous categorical variables was calculated using simple agreement (%) and kappa coefficient. Kappa coefficients are categorized by the following nomenclature: poor (κ < 0.00); slight (0.00 ≤ κ ≤ 0.20); fair (0.21 ≤ κ ≤ 0.40); moderate (0.41 ≤ κ ≤ 0.60); substantial (0.61 ≤ κ ≤ 0.80); and almost perfect (κ > 0.80) [31]. A *p*-value <0.05 was considered statistically significant. Statistical analyses were performed using STATA/IC 13.0 (StataCorp, Texas).

## Results

### Mixed HPV infections are frequent and unresolved by dideoxy (Sanger) sequencing

A total of 84 cytology samples were collected for this study. Twelve LSIL samples were excluded because of technical failure in the deep sequencing assay. Hence 72 samples categorized as LSIL (*n* = 43) and HSIL (*n* = 29) were analyzed and reported herein. The demographic distribution of the subjects (*n* = 72) whose cytological samples underwent study were as follows: White (43%), Black (11%), Asian (3%), other (22%), and unknown (21%). The median age of the cohort was 26 years (IQR, 23-33). For the HSIL group, the median age (28 years [IQR, 24-33]) was greater than that of the LSIL group (24 years [IQR, 23-31]) (median test, *p* = 0.02) (age distribution shown in Additional File 2: Figure S1). Histologic validation of the cytology samples showed overall good agreement (78%) (κ = 0.6, *p* <0.001) as summarized in Table 1.

**Table 1:** Cytohistological correlation and HPV E6/E7 deep sequencing results

Cytohistological Correlation										
Histology			Total			LSIL				HSIL
Samples, n			72			43				29
Histology (biopsy or excision)^a^										
Documented, n (%)			51 (71)			26 (60)				25 (86)
Not documented, n (%)			21(29)			17(40)				4(14)
Histological Grade^a^										
CIN 0, n (%)			4 (8)			4 (15)				0
CIN I, n (%)			23 (45)			19 (73)				4 (14)
CIN II/III, n (%)			24 (47)			3 (12)				21 ﻿(84)
Cytohistological agreement^b^										
Agreement, %			78							
Expected Agreement, %			46							
Kappa			0.6							
Std. Error			0.12							
*p*-value			<0.001							
			Deep Sequencing Reads for LSIL/HSIL							
	Merged^c^					Mapped^d^				
Read Statistic	Total	Total	HPV#1	HPV#2	HPV#3	HPV#4	HPV#5	HPV#6	HPV#7	HPV#8
Samples, n	72	72	72	44	25	13	8	5	2	1
Median, n	242,665	192,236	178,582	14,483	4,040	2,480	2,403	578	590	304
Minimum, n	11,174	6,022	6,022	367	135	407	412	366	332	-
Maximum, n	760,725	673,342	656,806	147,734	62,768	36,970	4,910	3,356	847	-
25th %tile, n	185,144	155,214	99,651	3,535	1,021	566	729	379	332	-
75th %tile, n	324,210	269,603	243,612	45,484	13,102	6,712	3,717	1,634	847	-
			HPV Genotype Concordance Analysis							
					Deep vs. Sanger sequencing^e^					
Agreement Statistic				LSIL				HSIL		
Samples, n				43				29		
Agreement, %				93				93		
Expected Agreement, %				13				53		
Kappa				0.92				0.85		
Std. Error				0.054				0.119		
p-value				<0.001				<0.001		

*CIN* cervical intraepithelial neoplasia, *E6/E7* HPV E6/E7 gene amplified by PCR, *HSIL* high-grade squamous intraepithelial lesion, *HPV* human papillomavirus, *LSIL* low-grade squamous intraepithelial lesion

^a^Cervical histopathology is based on the highest grade documented on cervical biopsy or therapeutic excisional biopsy, i.e. cold knife conization (CKC) and loop excisional procedure (LEEP). Absence or presence of pathology reports in the DoD electronic health records was categorized as “Documented” or “Not documented,” respectively

^b^Cytohistological agreement was calculated using samples with documented histopathology

^c^The total number of passed filtered reads was 21 million and merged reads was 18 million

^d^The mapped reads corresponding to the nth HPV genotype(s) found in LSIL and HSIL samples is listed in descending order

^e^The dominant (most abundant) HPV genotype determined by BLAST alignment of deep and dideoxy sequences were paired for concordance analysis

**Fig. 2:**
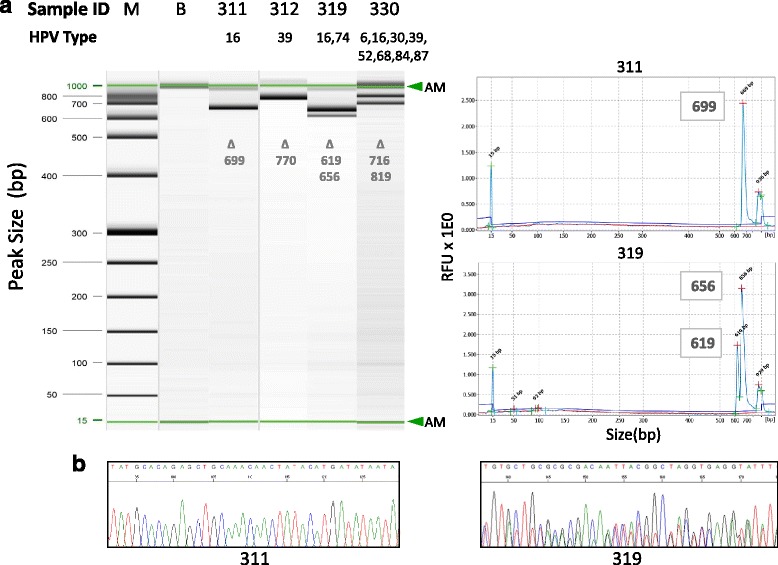
HPV DNA detection by PCR amplification, capillary electrophoresis and dideoxy (Sanger) sequencing. **a** Gel image and electropherogram of amplicon detection by high-resolution capillary electrophoresis. Representative samples #311, 312, 319, and 330 (HSIL) reveal 1 or 2 amplicons after using consensus primers (GP-E6/E7 F/B) to amplify an E6/E7 segment with an expected fragment size of ~660 bp (range, 619-819 bp). Amplicon size variability reflects sequence differences between HPV genotypes. In general, deep sequencing resolved a greater number of HPV genotypes than capillary electrophoresis per sample. Sample #330 illustrates this with detection of 2 amplicons on electrophoresis, but 8 genotypes by deep sequencing. **b** Representative sample (#311) with a single HPV infection revealing 1 amplicon (699 bp peak on electropherogram) and clean sequencing chromatogram. Representative sample (#319) with multiple HPV infections revealing 2 amplicons (619 and 656 bp peaks on electropherogram) and “noisy” overlapping peaks on the chromatogram. AM, alignment marker; B, buffer; bp, base pair; M, molecular-weight marker

The median concentration of extracted cellular DNAs was 43.5 ng/uL (IQR, 35.2-69.0). All DNA samples underwent PCR amplification of the HPV E6/E7 loci that yielded the expected 660-bp fragments on electrophoresis. Samples with single or multiple HPV infections displayed corresponding numbers of amplicon bands with detectable variations in base-pair size because of genotype-specific differences in the E6/E7 fragments (Fig. 2a). Multiple amplicons were detected in 25% (18/72) of specimens, with similar contributions from LSIL (12/43, 28%) and HSIL (6/29, 21%) (*χ* ^2^, *p* = 0.50). Downstream dideoxy sequencing of these amplicons revealed “clean” and “overlapping” patterns on the chromatogram indicative of pure and mixed E6/E7 sequences, respectively (Fig. 2b). Only the dominant HPV genotype within each sample that is resolvable by dideoxy sequencing is presented in Additional File 3: Table S2. These results were used for validation of genotyping by deep sequencing as described below.

### Deep sequencing of HPV E6/E7 loci reveals loss of HPV diversity and gain of clonal dominance in HSIL

The deep sequencing read statistics of all samples are summarized in Table 1. The total number of passed-filtered reads was 21 million, which is consistent with the maximum number of reads (25 million) for the MiSeq Reagent Kit used. The median number of merged reads for 72 samples derived from E6/E7 loci amplification was 242,665 (IQR, 185,144-324,210), and the proportion of mapped to merged reads was 79% (192,236/242,665). The number of mapped reads per sample was sufficient to discover up to 8 genotypes. The HPV genotypes based on BLAST^®^ are listed in Additional File 3: Table S2.

**Fig. 3:**
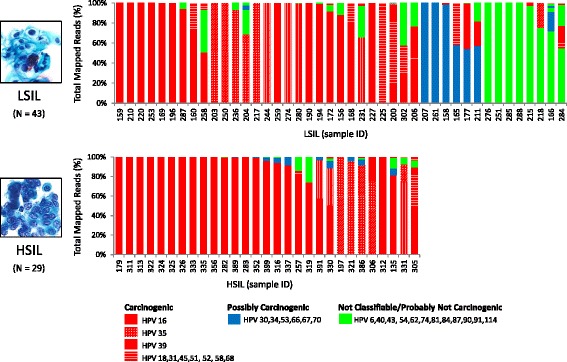
HPV genotype composition found in LSIL and HSIL samples. Deep sequencing of HPV E6/E7 amplicons derived from each LSIL or HSIL sample identified 1 to 8 HPV genotypes and quantitated their composition (%) based on number of mapped reads to total mapped reads. The top three dominant (highest proportion) genotypes found in LSIL were HPV-39, -16, and -35 [*red*, solid/hashed]. The carcinogenicity of LSIL dominant genotypes were: *carcinogenic* 29/43 (67%, *red*); *possibly carcinogenic* 6/43 (14%, *blue*); and *not classifiable/probably not carcinogenic* 8/43 (19%, *green*). For HSIL, the dominant genotype was primarily HPV-16 (21/29, 72%); and the dominant genotypes were all *carcinogenic* 29/29 (100%, *red*). The HPV carcinogenicity is based on IARC’s classification of human carcinogens [8]. HSIL, high-grade squamous intraepithelial lesion; IARC, International Agency for Research on Cancer; LSIL, low-grade squamous intraepithelial lesion; ID, identification

Figure 3 illustrates the HPV community found in LSIL and HSIL according to genotype and carcinogenicity. For LSIL E6/E7–amplicons (*n* = 43), deep sequencing identified the number of genotype(s)/sample as: 1(40%), 2 (26%), 3(21%), and ≥4 (14%). A total of 27 different genotypes were found in single and multi-infected samples. The dominant (most abundant) genotype in LSIL samples included: HPV-39, 11/43 (26%); -16, 9/43 (21%); and -35, 4/43 (9%). For HSIL E6/E7–amplicons (*n* = 29), deep sequencing identified the number of genotype(s)/sample as: 1(38%), 2(28%), 3(10%), and ≥4 (24%). Overall, 17 HPV different genotypes were identified in all HSIL samples. The dominant genotype in HSIL samples included: HPV-16, 21/29 (72%); -35, 4/29 (14%); and -39, 3/29 (10%). All dominant HPV genotypes found in HSIL were carcinogenic (29/29, 100%). In addition, the median age of the subjects who had a dominant, carcinogenic HPV genotype (26 years [IQR, 23–31]) versus all other IARC-defined categories (25.5 years [IQR, 24–38]) was not statistically different (median test, *p* = 0.77) (age distribution shown in Additional File 4: Figure S2).

**Fig. 4:**
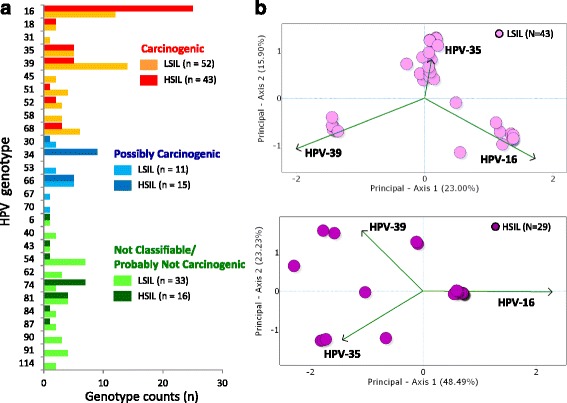
HPV diversity, dominance and community structure between LSIL and HSIL. **a** Bar chart represents the relative abundance of HPV genotypes found in LSIL and HSIL samples by deep sequencing. A total of 27 genotypes out of 43 samples were found in LSIL versus 17 genotypes out of 29 samples for HSIL with respective Shannon Wiener Indices, 3.01 and 2.28. The dominant (most abundant) genotype in LSIL was HPV-39 (BPI, 0.15) versus HPV-16 for HSIL (BPI, 0.34). *Species diversity* analysis between LSIL and HSIL revealed loss of HPV diversity (*, *p* <0.001) and domination by HPV-16 (*, *p* <0.001) in HSIL. **b** Principal component analysis (PCA) plots of HPV genotype composition in LSIL and HSIL samples. For LSIL and HSIL, HPV-16, 35, and 39 were identified as the three most influential genotypes within both HPV communities. Further species dissimilarity analysis revealed greater average abundance of HPV-16 (68%) and -35 (13%) for HSIL versus HPV-39 (24%) for LSIL. BPI, Berger-Parker Index; SWI, Shannon Wiener Index

HPV genotype diversity analysis (shown in Fig. 4a) based on the Shannon-Wiener Index (SWI) revealed a significant loss of genotype diversity from LSIL (SWI, 3.01) to HSIL (SWI, 2.28) (*p* <0.001) and domination by HPV-16 in HSIL (BPI = 0.34) (*p* <0.001). Diversity between HPV communities of LSIL and HSIL varied significantly (ANOSIM *R* = 0.07, *p* <0.05). We determined the three most influential HPV genotypes (-16, -35, -39) in the mixed compositions of LSIL and HSIL samples by PCA (Fig. 4b). Among these, species dissimilarity analysis determined a greater average abundance of HPV-16 (68%) and -35 (13%) in HSIL; in contrast, HPV-39 (24%) was more abundant in LSIL.

Validation of our next-generation genotyping results showed high concordance between the deep and dideoxy sequencing methods. Comparing the dominant HPV genotypes derived from the two methods, the inter-assay agreement was highly concordant for LSIL (κ = 0.91, *p* <0.001) and HSIL (κ = 0.85, *p* <0.001) (Table 1). This finding indicates that dideoxy sequencing may be used to determine the dominant genotype within mixed infections.

### Evolutionary relationship of HPV E6/E7 sequences correlate with carcinogenic potential

**Fig. 5:**
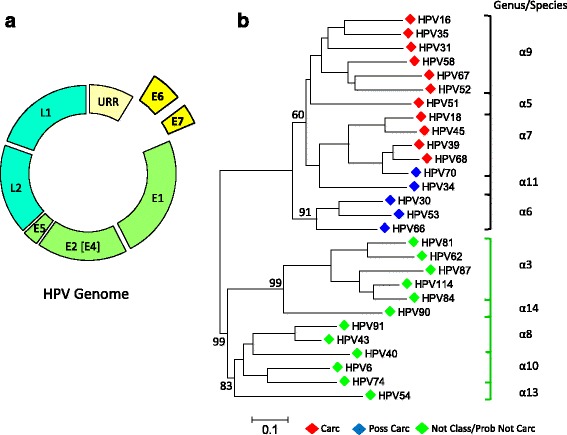
Evolutionary relationships of HPV E6/E7 sequences derived from LSIL and HSIL samples. **a** Prototypical HPV genome based on the genetic information of HPV-16. The contiguous E6 (477 bp) and E7 (297 bp) gene segment of each sample was the target used for sequencing, genotyping, and phylogenetic analysis. **b** Phylogenetic tree of 28 representative E6/E7 nucleotide sequences (one from each genotype) revealed two distinct clades: high-risk [*black* bracket] and low-risk [*green* bracket] that cluster respective species (α-5, 6, 7, 9, 11) and (α-3, 8, 10, 13, 15) within the α-genus. The evolutionary distances between species also correlate with the level of IARC-defined carcinogenicity. This finding is consistent with phylogenetic trees constructed traditionally from L1 ORF sequences [8]. The tree was inferred by the Neighbor-Joining method with the Maximum Composite Likelihood nucleotide substitution model using MEGA6 [29]. The scale bar indicates nucleotide substitutions per site. The bootstrap values are displayed for the primary and secondary nodes. The full tree is shown in Additional file 5: Figure S3. Carc, carcinogenic; Not Class, not classifiable; Poss Carc, possibly carcinogenic; Prob Not Carc, probably not carcinogenic

A neighbor joining tree was constructed from 160 E6/E7 sequences derived from single and mixed infections of 72 LSIL/HSIL samples (full tree shown in Additional file 5: Figure S3). A representative tree constructed from 28 E6/E7 sequences (one from each genotype) is presented in Fig. 5. The aligned E6/E7 sequences grouped likewise to L1-based phylogenetic trees [8, 30]. Moreover, the genetic distances or evolutionary divergences between the species correlated with IARC-defined carcinogenicity [8, 30]. Taken together, the genetic sequence of E6/E7 alone was sufficient to genotype and phenotype (carcinogenicity) the samples. Another observation is the non-detectable difference between E6/E7 branch lengths for LSIL and HSIL among individual genotypes. This finding suggests that disease severity was not associated with HPV-subtype differences (2–10% nucleotide differences) [6].

## Discussion

**Fig. 6:**
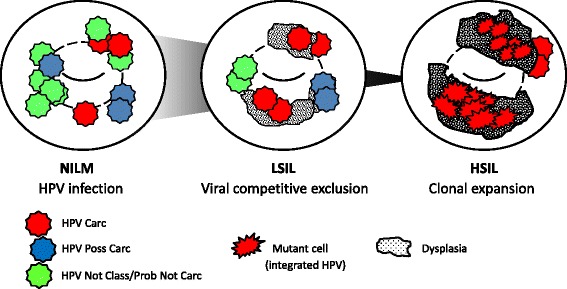
Schematic diagram of evolving virus-host interactions on the cervix. After infection (left), virus-virus (middle) and virus-host (right) interactions based on the principles of competitive exclusion and clonal evolution of cancer are illustrated [34–40]. Gause’s law of competitive exclusion states that between two competing species, the species with the slightest advantage will ultimately dominate [34, 35]. The deep sequencing results of this study corroborated this phenomenon in the virome with loss of diversity and gain of dominance by carcinogenic HPVs as LSIL progressed to HSIL. Furthermore, the predominantly monotypic, carcinogenic virome observed in HSIL may be attributed to monoclonal expansion of host cells with genome-integrated HPV DNA [36–38]. Carc, carcinogenic; HPV, Human papillomavirus; Not Class, not classifiable; Poss Carc, possibly carcinogenic; Prob Not Carc, probably not carcinogenic

This study revealed the complex HPV communities residing in abnormal cervical cytology. We found that patients with LSIL and HSIL are frequently (~60%) infected with multiple HPV genotypes. Deep amplicon sequencing generated abundant mapped reads and deciphered the composition of genotypes within each sample. The total number of HPV types identified by sequencing single and multi-infected samples ranged from 27 for LSIL to 17 for HSIL and spanned the spectrum of IARC-defined carcinogenicity [8, 30]. More specifically, the viral community differed between LSIL and HSIL with a loss of genotypic diversity and domination by carcinogenic HPVs, in particular, HPV-16 in HSIL. The inverse correlation between HPV diversity and progressive disease is consistent with the findings of 1,518 cervical biopsies ranging from CIN 0 to 3 in the ATHENA (Addressing The Need for Advanced HPV diagnostics) trial [32]. Furthermore, carcinogenic HPVs, in particular HPV-16 and -18, have been shown to be indicators and predictors of CIN 3 development [32, 33]. Carcinogenic HPV dominance (≥50%) may also be an indicator of underlying high-grade disease, as found in 12% (3/26) of LSIL upgraded to CIN 2/3 on biopsy. Together, these metagenomic characteristics are consistent with the ecological principles of competitive exclusion and carcinogenesis hallmarked by clonal expansion and evolution of transformed cells as illustrated in Fig. 6 [34–40]. The distinguishing features of altered diversity and dominance between HPV communities may serve well as a biomarker for disease severity. The addition of evolutionary analysis to sample E6/E7 sequences assists in determining carcinogenic potential based on genetic distances to HPV reference sequences. In this way, deep sequencing revealed the dynamic ecology of HPV coexisting and evolving within the cervical epithelium, a characteristic that would otherwise remain unseen by traditional sequencing.

Currently, published data on deep sequencing of HPV in abnormal cytology are limited and varied. Previous studies have analyzed target (non-generalizable) populations, used different NGS platforms and assays, and reported findings that may not be translatable between platforms [13–16, 41]. However, we did find several notable similarities. A metagenomics study of healthy persons from the NIH Human Microbiome Project revealed a high prevalence of HPV in the vagina (41.5%) with 43 types; high abundance of HPV-34, 53, 45, and 52; and a high rate (~50%) of mixed infections [41]. Fonseca et al. investigated the prevalence of HPV in isolated, indigenous Amazonian women of northern Brazil [15]. Among the 607 cytology samples, 3.3% were abnormal, which included 7 cases of LSIL, 2 cases of HSIL, and 1 case of carcinoma. The overall HPV prevalence was 39.7% with 60 different genotypes and a high rate (45%) of multiple infections. Only a limited number of HPVs were found in HSIL and/or carcinoma (HPV-16 and 31) and ASC-US/LSIL (HPV-16, 18, and 31). Finally, Meiring and colleagues used deep sequencing to identify 16 HPV types in a South African HIV+ woman and demonstrated that prevalent HPV types in HIV+ women are undetectable by commercial tests that complicate surveillance measures [16]. Taken together, these data demonstrate that NGS reveals a diverse and prevalent existence of mixed HPV infections in healthy females. With progression of cytopathology, diversity diminishes to a few virulent types, namely HPV-16 and other α-7, -9 species as observed in our LSIL/HSIL samples and Sjoeborg’s study using linear array genotyping [42]. Finally, for immunocompromised hosts, the HPV virome may be more diverse and inclusive of less-virulent genotypes.

In regards to age, our subjects with LSIL were younger than those with HSIL. This finding is consistent with the natural history of HPV infection with a characteristic peak age of < 25 years for HPV infection/LSIL and 25–35 years for HSIL [43]. Ironically, we found no difference in the median age of subjects between those who had and who did not have carcinogenic HPV genotypes by NGS. A large population-based study conducted in New Mexico showed similar age distributions as our sample for both carcinogenic and low-risk HPV groups with the peak age range being 21–24 years followed by a rapid decline [44]. In fact, over 35% of women <21 years of age and 32% between the ages of 21–24 years tested positive for any carcinogenic HPV on cytology [44]. Collectively, the disparate age distributions support the notion that infection with a carcinogenic HPV occurs predominantly in adolescence/early adulthood; whereas, disease, i.e. HSIL develops in later adulthood.

The strength of this investigation lies in the clinical specimens studied and sequencing method used. First, we intentionally focused on precancerous cervical lesions, i.e. LSIL and HSIL because they have been understudied to date by deep sequencing. The remarkable genotypic diversity and coinfection rates found in this study have direct implications for vaccine development and epitope selection, as well as post-immunization surveillance for efficacy and emerging replacement-types [45]. The recently FDA-approved 9-Valent HPV vaccine (9vHPV) containing HPV 6, 11, 16, 18, 31, 33, 45, 52, and 58 virus-like particles (VLPs) is a significant improvement over the quadrivalent vaccine. However, our study only found 7/27 and 4/17 genotypes (~25%) in LSIL and HSIL, respectively, covered by the 9vHPV epitopes. More importantly, HPV-35 and -39 found highly prevalent in LSIL and HSIL are not covered. Another additive concern about vaccine ineffectiveness is the low vaccine uptake and adherence rates in the U.S. [46, 47]. Consequently, active, population-based surveillance is necessary to detect potential redistribution of carcinogenic HPVs as a result of under-achieved herd immunity [45, 48–50]. Second, we used the most accurate NGS platform commercially available [12]. The least cumbersome DNA library preparation was chosen to streamline the laboratory workflow. The bioinformatics workflow created for HPV genotyping with automatable steps systematized the computational analysis. The simple methods developed and tested in this study may be adopted for studying HPV viromes at all susceptible anatomical sites where surveillance is essential [50]. Conversely, the high concordance rate found between deep and Sanger sequencing suggests that Sanger remains a reliable method of detecting the dominant genotype in single and mixed infections if decipherable by BLAST®. In resource limited settings, Sanger sequencing will remain salient. Although PCR/Sanger sequencing is still the current reference standard for HPV clinical diagnostics, next-generation genotyping may soon gain acceptance in the clinical realm as a new reference method according to the 2015 FDA Draft Guidance on HPV in vitro diagnostic devices [51]. Before adoption, however, we opine that further investigation is required to determine the threshold for variant detection (minimum coverage) and variant filtering (percentage of reads in a mixed population) for accurate next-generation HPV genotyping. Two independent studies using admixtures of Hepatitis C Virus (HCV) and influenza A plasmids suggest that for accurate detection using Illumina’s sequencing technology, the limit of variant detection and filtering should be set ~100x and 0.5%, respectively [52, 53].

Cross-sectional studies, similarly to other observational studies are susceptible to errors due to chance and bias. We acknowledge that the current study has limitations. First, the potential for selection bias must be considered. Our clinical samples were derived from a South Texas military population represented largely by Active Duty Members and dependents of the U.S. Army and Air Force. The demographics, social, and sexual behavior of our population may not be representative of other segments of the U.S. population. In general, HPV prevalence should be interpreted in the context of ethnogeography. Second, atypical squamous and glandular cells of undetermined significance (ASC-US and AGUS) cytological categories were not studied. The overall frequency of HPV+/ASC-US (1.1%) and HPV+/AGUS (0.05%) is low among screening cytology samples; however, the 5-year risk of histologic HSIL and cancer are significant, i.e. 18 and 45%, respectively [54]. Hence, to fill this knowledge gap, we plan to investigate HPV metagenomes of uncommon cytological categories to further our understanding of viral ecology, and we plan to establish predictive models for cytological outcomes based on metagenomics profiles. Third, we did not study LSIL/HSIL samples with negative E6/E7 amplification results. PCR non-detection (false-negative results) may be attributed to several variables, e.g. insufficient DNA template quantity or quality and primer-target mismatch [11, 55]. To explore and compare the HPV metagenomes in E6/E7 amplicon-positive and -negative cytology, multiply-primed rolling-circle amplification followed by deep sequencing may offer a solution, in particular, to partially deleted or poorly E6/E7-primed HPV genomes [13].

## Conclusions

Deep sequencing has provided a powerful lens through which to peer into viral communities and gain an understanding of a dynamic microcosm imperceptible with conventional methods. The HPV diversity and community characteristics found in LSIL and HSIL have provided vital information relevant to cervical carcinogenesis, biomarker discovery, vaccinology, and surveillance strategies. With revolutionary advances in sequencing and computational technologies, we are now able to decipher and interpret the cryptic codes of an ancient virus in a manner reminiscent of the Shakespearean metaphor, “In nature’s infinite book of secrecy, a little I can read” [56].

## Additional files

Additional file 1:
**Table S1.** CLC Genomics workflow settings for next-generation HPV genotyping. (PDF 129 kb)
Additional file 2:
**Figure S1.** Age distribution by LSIL and HSIL cytological grades. Age distribution of the sample population according to cytological grade. The median age (24 years [IQR, 23–31]) of the LSIL group (*N* = 43) was younger than the median age (28 years [IQR, 24–33]) of the HSIL group (*N* = 29) (median test, *p* = 0.02). *Abbreviations:* HSIL, high-grade squamous intraepithelial lesion; LSIL, low-grade squamous intraepithelial lesion. *Notations:* Median (vertical line), Gaussian kernel density estimate (dashed curve). (PDF 236 kb)
Additional file 3:
**Table S2.** HPV E6/E7 sequencing: dideoxy (Sanger) vs. deep (Illumina). (PDF 236 kb)
Additional file 4:
**Figure S2.** Age distribution by HPV carcinogenic potential. Age distribution of the sample population according to HPV carcinogenicity. The median age of the subjects who had a dominant, carcinogenic HPV genotype (26 years [IQR, 23-31]) versus all other IARC-defined categories (25.5 years [IQR, 24–38]) was not statistically different (median test, *p* = 0.77). *Abbreviations:* IARC: International Agency for Research on Cancer. *Notations:* Median (vertical line), Gaussian kernel density estimate (dashed curve). (PDF 242 kb)
Additional file 5:
**Figure S3.** Evolutionary relationships of E6/E7 sequences derived from LSIL and HSIL. The phylogenetic tree revealed the 3 distinct clades (*) that cluster unique species i.e., (α-5, 7, 9), (α-6), and (α-3, 8, 10, 13, 15) within the α-genus. Additionally, the evolutionary distances between the 3 clades correlated with IARC defined carcinogenicity. This finding is consistent with phylogenetic trees constructed traditionally from L1 ORF sequences. The evolutionary history was inferred using the Neighbor-Joining method [24]. The optimal tree with the sum of branch length = 7.80202397 is shown. The percentage of replicate trees in which the associated taxa clustered together in the bootstrap test (1,000 replicates) are shown next to the branches [25]. The tree is drawn to scale, with branch lengths in the same units as those of the evolutionary distances used to infer the phylogenetic tree. The evolutionary distances were computed using the Maximum Composite Likelihood method [25] and are in the units of the number of base substitutions per site. The analysis involved 160 nucleotide sequences. Codon positions included were 1st + 2nd + 3rd + Noncoding. All positions containing gaps and missing data were eliminated. There were a total of 238 positions in the final dataset. Evolutionary analyses were conducted in MEGA6 [27]. (PDF 431 kb)

## Abbreviations

AGUS: Atypical glandular cells of undetermined significance
ANOSIM: Analysis of similarity
ASC-US: Atypical squamous cells of undetermined significance
BLAST: Basic local alignment search tool
BPI: Berger-Parker Index
CIN: Cervical intraepithelial neoplasia
HPV: Human papillomavirus
HSIL: High-grade squamous intraepithelial lesion
HTS: High-throughput sequencing
IARC: International Agency for Research on Cancer
LSIL: Low-grade squamous intraepithelial lesion
NGS: Next-generation sequencing
NILM: Negative for intraepithelial lesion or malignancy
Pap: Papanicolaou smear
PCA: Principal component analysis
SWI: Shannon-Wiener Index
TS: Trace score
VLP: Virus-like particles.
