# Supplementary material for: Deep sequencing of HPV E6/E7 genes reveals loss of genotypic diversity and gain of clonal dominance in high-grade intraepithelial lesions of the cervix
Source: BMC Genomics. 2017 Mar 14;18:231. doi: 10.1186/s12864-017-3612-y (PMC5348809; doi:10.1186/s12864-017-3612-y)
Supplement: Additional file 1: — Table S1. CLC Genomics workflow settings for next-generation HPV genotyping. (PDF 129 kb) [file 12864_2017_3612_MOESM1_ESM.pdf]

Table S1

CLC Genomics workflow settings for next-generation HPV genotyping

Sequential steps	Options	Selection or Input
Import paired-end data		
General Options	Paired reads	Checkbox (checked)
	Discard read names	Checkbox (checked)
	Paired end (forward-reverse)	Checkbox (checked)
	Minimum distance	150
	Maximum distance	500
Illumina Options	Remove failed reads	Checkbox (checked)
	MiSeq de-multiplexing	Checkbox (checked)
	Quality scores	NCBI/Sanger or Illumina Pipeline 1.8 or later
Trim Sequences		
Quality trimming	Trim using quality scores	Checkbox (checked); Limit: 0.05
	Trim ambiguous nucleotides	Checkbox (checked); Maximum number: 2
Adapter trimming	Import file	Nextera XT indices (Trim adapter list (.xlsx))
	Search on both strands	Checkbox (checked)
Sequence filtering	Discard reads below length	Checkbox (checked):15
De novo Assembly		
De novo Options	Automatic word size	Checkbox (unchecked); Word size: 45 (default)
	Automatic bubble size	Checkbox (unchecked); Bubble size: 98 (default)
	Contig length	Minimum contig length: 200
	Auto-detect paired distances	Checkbox (unchecked)
	Perform scaffolding	Checkbox (checked)
Mapping Options	Map reads back to contigs	Radio button (on)
	Mismatch cost	2 (default)
	Insertion cost	3 (default)
	Deletion cost	3 (default)
	Length fraction	0.5 (default)
	Similarity fraction	0.8 (default)
	Global alignment	Checkbox (checked)
	Update contigs	Checkbox (checked)
	Create list of un-mapped reads	Checkbox (checked)
	Extract Subset	Top contigs with total read count $\geq$ 100
Multi-BLAST at NCBI		
Program and database	Program	Dropdown: blastn: DNA sequence and database
	Database	Dropdown: Nucleotide collection (nr)
BLAST parameters	Limit by entrez query	Dropdown: Viruses [ORGN]
	Filter low complexity	Checkbox (checked)
	Expect	1e-10
	Word size	10
	Match/Mismatch	Match 2; Mismatch -3
	Gap costs	Existence 5, Extension 2
	Maximum no. of hit sequences	50
