# Supplementary figures and images for: Deep sequencing of HPV E6/E7 genes reveals loss of genotypic diversity and gain of clonal dominance in high-grade intraepithelial lesions of the cervix
Source: BMC Genomics. 2017 Mar 14;18:231. doi: 10.1186/s12864-017-3612-y (PMC5348809; doi:10.1186/s12864-017-3612-y)

Fig. S1

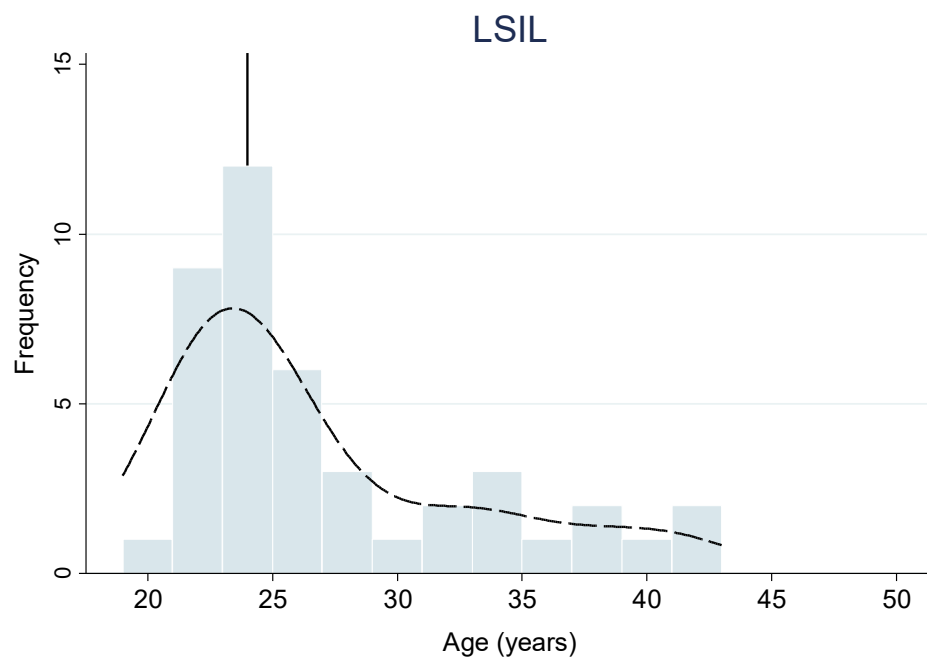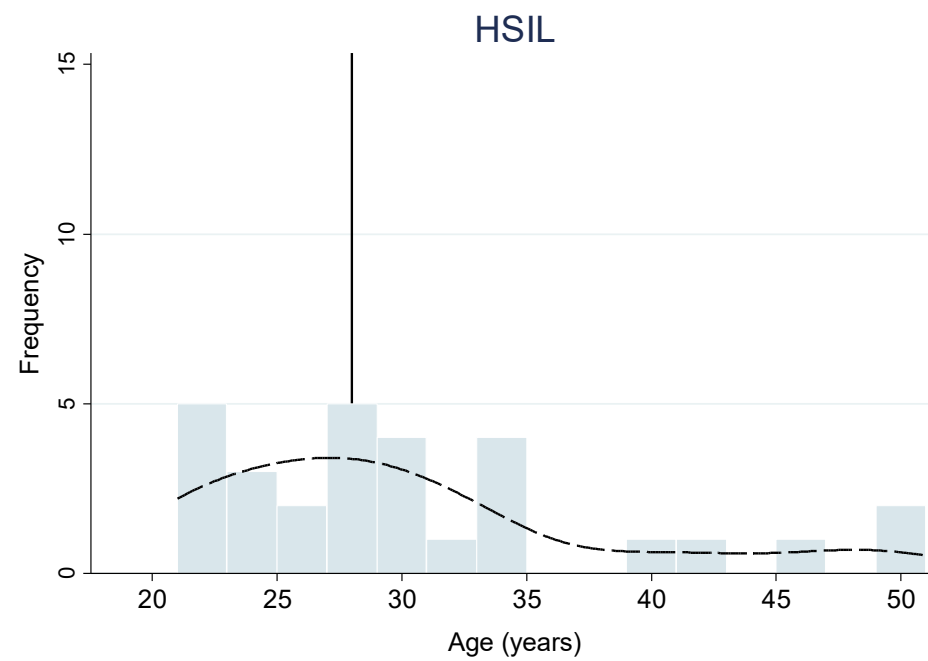

Supplement: Additional file 2: — Figure S1. Age distribution by LSIL and HSIL cytological grades. Age distribution of the sample population according to cytological grade. The median age (24 years [IQR, 23–31]) of the LSIL group (N = 43) was younger than the median age (28 years [IQR, 24–33]) of the HSIL group (N = 29) (median test, p = 0.02). Abbreviations: HSIL, high-grade squamous intraepithelial lesion; LSIL, low-grade squamous intraepithelial lesion. Notations: Median (vertical line), Gaussian kernel density estimate (dashed curve). (PDF 236 kb) [file 12864_2017_3612_MOESM2_ESM.pdf]

Fig. S2

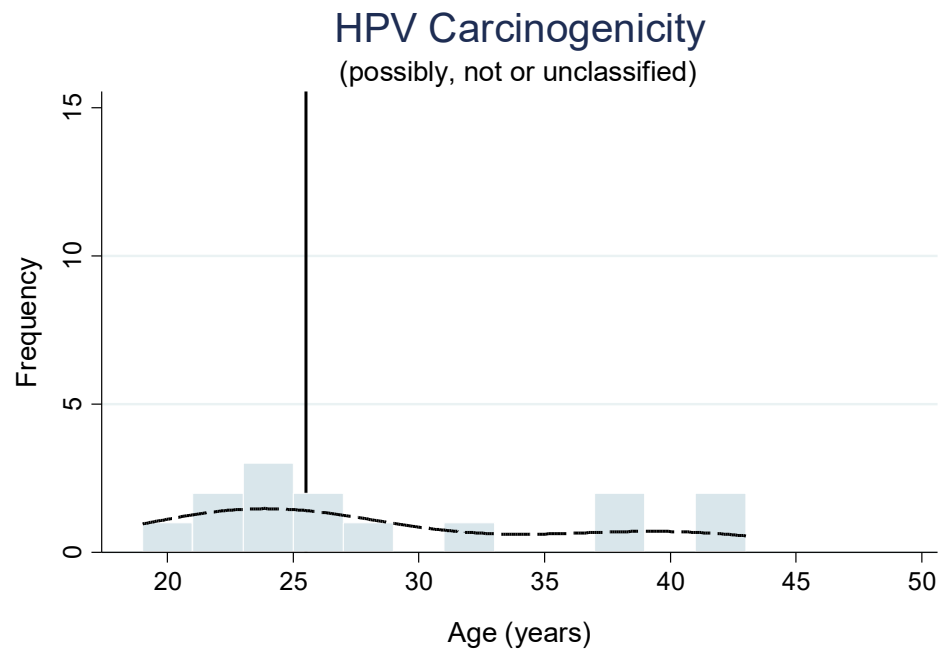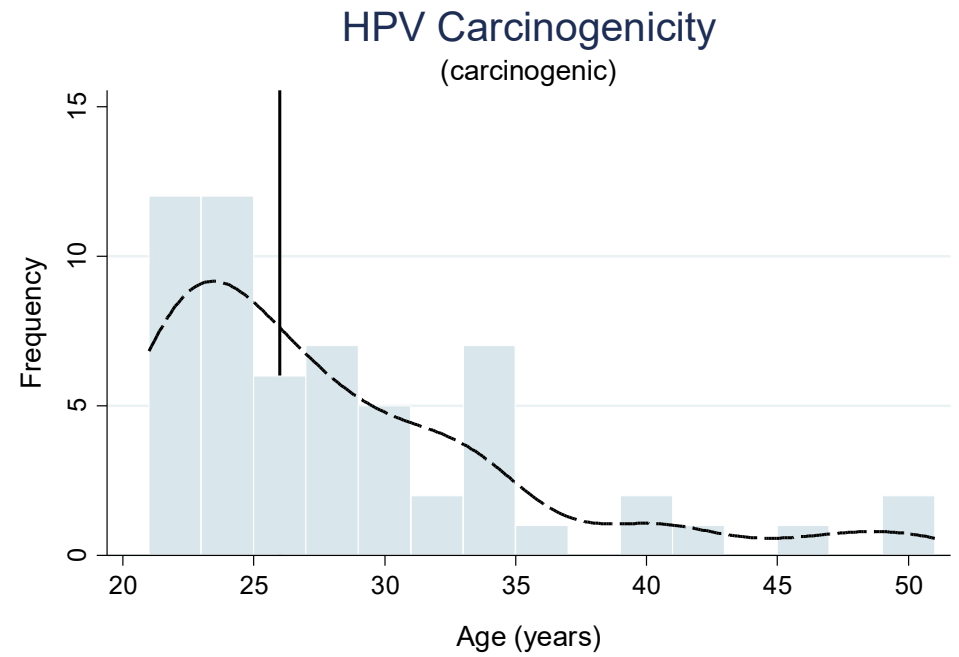

Supplement: Additional file 4: — Figure S2. Age distribution by HPV carcinogenic potential. Age distribution of the sample population according to HPV carcinogenicity. The median age of the subjects who had a dominant, carcinogenic HPV genotype (26 years [IQR, 23-31]) versus all other IARC-defined categories (25.5 years [IQR, 24–38]) was not statistically different (median test, p = 0.77). Abbreviations: IARC: International Agency for Research on Cancer. Notations: Median (vertical line), Gaussian kernel density estimate (dashed curve). (PDF 242 kb) [file 12864_2017_3612_MOESM4_ESM.pdf]

Fig. S3

Genus/Species

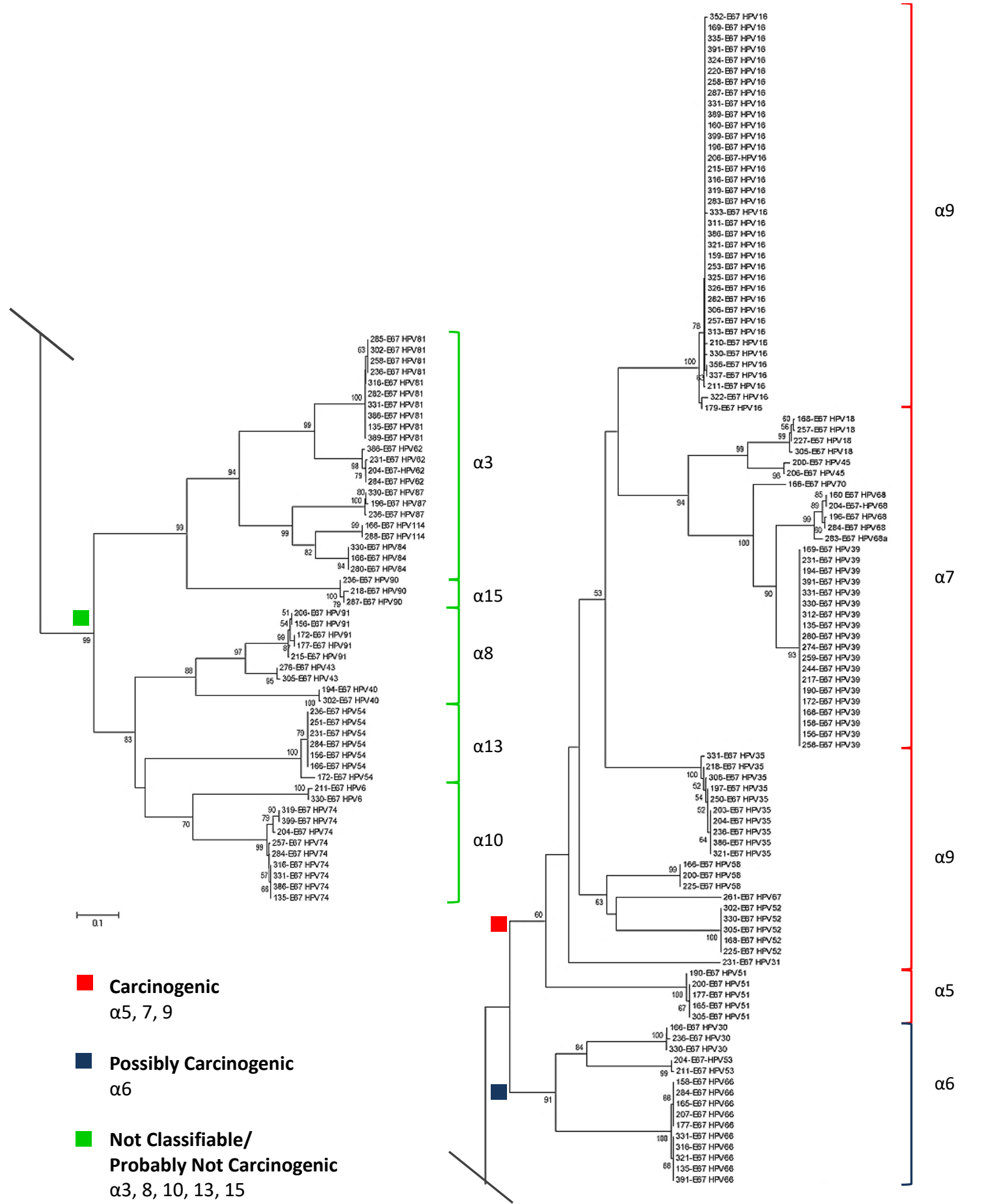

Supplement: Additional file 5: — Figure S3. Evolutionary relationships of E6/E7 sequences derived from LSIL and HSIL. The phylogenetic tree revealed the 3 distinct clades (*) that cluster unique species i.e., (α-5, 7, 9), (α-6), and (α-3, 8, 10, 13, 15) within the α-genus. Additionally, the evolutionary distances between the 3 clades correlated with IARC defined carcinogenicity. This finding is consistent with phylogenetic trees constructed traditionally from L1 ORF sequences. The evolutionary history was inferred using the Neighbor-Joining method [24]. The optimal tree with the sum of branch length = 7.80202397 is shown. The percentage of replicate trees in which the associated taxa clustered together in the bootstrap test (1,000 replicates) are shown next to the branches [25]. The tree is drawn to scale, with branch lengths in the same units as those of the evolutionary distances used to infer the phylogenetic tree. The evolutionary distances were computed using the Maximum Composite Likelihood method [25] and are in the units of the number of base substitutions per site. The analysis involved 160 nucleotide sequences. Codon positions included were 1st + 2nd + 3rd + Noncoding. All positions containing gaps and missing data were eliminated. There were a total of 238 positions in the final dataset. Evolutionary analyses were conducted in MEGA6 [27]. (PDF 431 kb) [file 12864_2017_3612_MOESM5_ESM.pdf]
