# Supplementary material for: Deep sequencing of HPV E6/E7 genes reveals loss of genotypic diversity and gain of clonal dominance in high-grade intraepithelial lesions of the cervix
Source: BMC Genomics. 2017 Mar 14;18:231. doi: 10.1186/s12864-017-3612-y (PMC5348809; doi:10.1186/s12864-017-3612-y)
Supplement: Additional file 3: — Table S2. HPV E6/E7 sequencing: dideoxy (Sanger) vs. deep (Illumina). (PDF 236 kb) [file 12864_2017_3612_MOESM3_ESM.pdf]

Table S2

HPV E6/E7 sequencing: dideoxy (Sanger) vs. deep (Illumina)

Sample Info and PCR[a]				Dideoxy Seq[b]		Deep Sequencing[c]			Top 4 HPV genotypes[d]							
No.	ID	PAP	PCR Band (n)	HPV Type	IARC Carc	Total Merged Reads (n)	Total Mapped Reads (n)	Total HPV Types (n)	HPV#1	HPV#1 Mapped Reads (n)	HPV#2	HPV#2 Mapped Reads (n)	HPV#3	HPV#3 Mapped Reads (n)	HPV#4	HPV#4 Mapped Reads (n)
1	159_E6/7	LSIL	1	**16**	CARC	265283	256807	1	**16**	256807						
2	210_E6/7	LSIL	1	**16**	CARC	211911	211366	1	**16**	211366						
3	220_E6/7	LSIL	1	**16**	CARC	189893	188077	1	**16**	188077						
4	253_E6/7	LSIL	1	**16**	CARC	205061	201803	1	**16**	201803						
5	169_E6/7	LSIL	1	**16**	CARC	100831	99307	2	**16**	98723	**39**	584				
6	196_E6/7	LSIL	1	**16**	CARC	196182	193973	3	**16**	189690	**68**	3784	**87**	499		
7	287_E6/7	LSIL	2	**16**	CARC	698914	673342	2	**16**	635823	**90**	37519				
8	160_E6/7	LSIL	2	**16**	CARC	80496	80241	2	**16**	59079	**68**	21162				
9	258_E6/7	LSIL	3	**81**	NC	184645	182700	3	**16**	92665	**81**	76933	**39**	13102		
10	203_E6/7	LSIL	1	**35**	CARC	181931	180739	1	**35**	180739						
11	250_E6/7	LSIL	1	**35**	CARC	182958	175736	1	**35**	175736						
12	236_E6/7	LSIL	2	**35**	CARC	80315	79179	6[d]	**35**	72748	**90**	3285	**81**	1789	**54**	566
13	204_E6/7	LSIL	3	**35**	CARC	112314	110654	5[d]	**35**	75908	**62**	16694	**68**	10542	**53**	4955
14	217_E6/7	LSIL	1	**39**	CARC	82821	82401	1	**39**	82401						
15	244_E6/7	LSIL	1	**39**	CARC	291207	290055	1	**39**	290055						
16	259_E6/7	LSIL	1	**39**	CARC	51251	50861	1	**39**	50861						
17	274_E6/7	LSIL	1	**39**	CARC	237497	223580	1	**39**	223580						
18	280_E6/7	LSIL	1	**39**	CARC	364539	363498	2	**39**	363131	**84**	367				
19	190_E6/7	LSIL	1	**39**	CARC	159203	158970	2	**39**	158513	**51**	457				
20	194_E6/7	LSIL	1	**39**	CARC	188780	187844	2	**39**	186123	**40**	1721				
21	172_E6/7	LSIL	2	**39**	CARC	205322	204422	3	**39**	188965	**54**	12272	**68**	3185		
22	156_E6/7	LSIL	2	**39**	CARC	347345	345857	4	**39**	303347	**91**	39431	**54**	1170	**68**	1909
23	168_E6/7	LSIL	1	**39**	CARC	210814	207242	3	**39**	165519	**18**	35650	**52**	6073		
24	231_E6/7	LSIL	3	**39**	CARC	213242	205876	4	**39**	135671	**62**	44771	**54**	18722	**31**	6712
25	227_E6/7	LSIL	1	**18**	CARC	244946	241575	1	**18**	241575						
26	225_E6/7	LSIL	1	**58**	CARC	246932	242293	2	**58**	230070	**52**	12223				
27	200_E6/7	LSIL	1	**51**	CARC	278800	259530	3	**51**	245648	**58**	11192	**45**	2690		
28	302_E6/7	LSIL	2	**52**	CARC	206871	163278	3	**52**	94851	**81**	67910	**40**	517		
29	206_E6/7	LSIL	1	**45**	CARC	272402	271880	3	**45**	121870	**16**	87242	**91**	62768		
30	207_E6/7	LSIL	1	**66**	POSC	246959	239254	1	**66**	239254						
31	261_E6/7	LSIL	1	**67**	POSC	173582	168336	1	**67**	168336						
32	158_E6/7	LSIL	1	**66**	POSC	279386	260597	2	**66**	255611	**39**	4986				
33	165_E6/7	LSIL	1	**51**	CARC	207267	203625	2	**66**	117879	**51**	85746				
34	177_E6/7	LSIL	1	**66**	POSC	72068	64680	3	**66**	34963	**51**	29582	**91**	135		
35	211_E6/7	LSIL	3	**53**	POSC	159126	151457	3	**53**	87245	**16**	36427	**6**	27785		
36	276_E6/7	LSIL	1	**43**	NC	168956	166828	1	**43**	166828						
37	251_E6/7	LSIL	1	**54**	NC	195217	190499	1	**54**	190499						
38	285_E6/7	LSIL	1	**81**	NC	34601	29487	1	**81**	29487						
39	288_E6/7	LSIL	1	**114**	NC	470073	445190	1	**114**	445190						
40	215_E6/7	LSIL	1	**91**	NC	185643	174089	2	**91**	169568	**16**	4521				
41	218_E6/7	LSIL	1	**90**	NC	358998	267325	2	**90**	199829	**35**	67496				
42	166_E6/7	LSIL	2	**114**	NC	294355	284775	6[d]	**114**	203991	**30**	56197	**84**	10732	**70**	5589
43	284_E6/7	LSIL	3	**74**	NC	192171	180116	5[d]	**62**	50240	**54**	47932	**68**	41038	**74**	36970
1’	179_E6/7	HSIL	1	**16**	CARC	209757	206925	1	**16**	206925						
2’	311_E6/7	HSIL	1	**16**	CARC	73918	65705	1	**16**	65705						
3’	313_E6/7	HSIL	1	**16**	CARC	240384	176425	1	**16**	176425						
4’	322_E6/7	HSIL	1	**16**	CARC	247599	92493	1	**16**	92493						
5’	324_E6/7	HSIL	1	**16**	CARC	318145	285040	1	**16**	285040						
6’	325_E6/7	HSIL	1	**16**	CARC	398221	378636	1	**16**	378636						
7’	326_E6/7	HSIL	1	**16**	CARC	24769	166837	1	**16**	166837						
8’	333_E6/7	HSIL	1	**16**	CARC	11174	6022	1	**16**	6022						
9’	335_E6/7	HSIL	1	**16**	CARC	286686	211676	1	**16**	211676						
10’	356_E6/7	HSIL	1	**16**	CARC	330274	319481	1	**16**	319481						
11’	282_E6/7	HSIL	1	**16**	CARC	366539	353150	2	**16**	352665	**81**	485				
12’	389_E6/7	HSIL	1	**16**	CARC	760725	658382	2	**16**	656806	**34**	1576				
13’	283_E6/7	HSIL	1	**16**	CARC	259524	174026	2	**16**	172854	**68**	1172				
14’	352_E6/7	HSIL	1	**16**	CARC	367538	284116	2	**16**	281965	**34**	2151				
15’	399_E6/7	HSIL	2	**16**	CARC	369582	178169	3	**16**	171240	**34**	6628	**74**	301		
16’	316_E6/7	HSIL	1	**16**	CARC	433128	180715	4	**16**	170076	**34**	9156	**66**	1021	**74**	462

17’	337_E6/7	HSIL	1	**16**	CARC	462102	114642	2	**16**	104747	**34**	9895				
18’	257_E6/7	HSIL	2	**16**	CARC	256624	123955	3	**16**	100578	**74**	17671	**18**	5706		
19’	319_E6/7	HSIL	2	**74**	NC	346552	338223	2	**16**	250913	**74**	87310				
20’	391_E6/7	HSIL	2	**16**	CARC	302384	118063	3	**16**	67826	**39**	46197	**66**	4040		
21’	330_E6/7	HSIL	2	**16**	CARC	432006	389058	8[d]	**16**	195884	**39**	147734	**30**	31029	**6**	9185
22’	197_E6/7	HSIL	1	**35**	CARC	429751	420694	1	**35**	420694						
23’	321_E6/7	HSIL	1	**35**	CARC	264315	115364	4	**35**	110537	**34**	3837	**66**	499	**16**	491
24’	386_E6/7	HSIL	1	**35**	CARC	187497	64171	5[d]	**35**	58766	**34**	3792	**81**	651	**74**	407
25’	306_E6/7	HSIL	2	**35**	CARC	398660	380652	2	**35**	285594	**16**	95058				
26’	312_E6/7	HSIL	1	**39**	CARC	309676	291254	2	**39**	289850	**68**	1404				
27’	135_E6/7	HSIL	1	**58**	CARC	191642	44035	6[d]	**39**	35819	**34**	3129	**54**	2230	**81**	1589
28’	331_E6/7	HSIL	1	**39**	CARC	407890	169059	7[d]	**39**	125071	**35**	31866	**34**	5425	**81**	2480
29’	305_E6/7	HSIL	1	**51**	CARC	253012	246747	4	**51**	201831	**52**	18638	**43**	16951	**18**	9327

CARC, carcinogenic HPV; E6/7, HPV E6/E7 gene amplified by PCR; HSIL, high-grade squamous intraepithelial lesion; HPV, human papillomavirus; ID, sample identification; IARC Carc, International Agency for Research on Cancer - classification of carcinogenicity; L1, HPV L1 gene amplified by PCR; LSIL, low-grade squamous intraepithelial lesion; PCR, polymerase chain reaction; POSC, possibly carcinogenic; NC, not classifiable/probably not carcinogenic; No., sequentially numbered samples grouped as LSIL or HSIL and denoted by apostrophe; PAP, Pap smear; Seq, sequencing.

[a] Cytologically derived DNA samples amplified by PCR using consensus primers to target the HPV E6/E7 loci. The number of PCR amplicon bands was determined by high-resolution capillary gel electrophoresis.

[b] HPV genotype determined by BLAST alignment after amplicon sequencing (dideoxy method). HPV genotype number in bold.

[c] HPV genotype determined by BLAST alignment after amplicon sequencing (deep method). HPV genotype number in bold.

[d] Up to 4 most abundant HPV genotype(s) per sample are tabulated. For 8 samples with > 4 types, the additional genotypes (each constituting < 2% of the HPV composition within a sample) are listed as follows: 135_E6/7 (HPV-66,-74); 166_E6/7 (HPV-54,-58); 204_E6/7 (HPV-74); 236_E6/7 (HPV-30,-87); 284_E6/7 (HPV-66); 330_E6/7 (HPV-52,-68,-84,-87); 331_E6/7 (HPV-16,-66,-74); 386_E6/7 (HPV-16).
